# *N*-(2,3-Dimethyl­phen­yl)-2,4-dimethyl­benzene­sulfonamide

**DOI:** 10.1107/S1600536810011669

**Published:** 2010-04-02

**Authors:** P. G. Nirmala, B. Thimme Gowda, Sabine Foro, Hartmut Fuess

**Affiliations:** a Department of Chemistry, Mangalore University, Mangalagangotri 574 199, Mangalore, India; b Institute of Materials Science, Darmstadt University of Technology, Petersenstrasse 23, D-64287 Darmstadt, Germany

## Abstract

The asymmetric unit of the title compound, C_16_H_19_NO_2_S, contains two independent mol­ecules: the dihedral angles between the sulfonyl and anilino benzene rings in the two mol­ecules are 41.5 (1) and 43.8 (1)°. The independent mol­ecules are linked into a dimer by a pair of inter­molecular N—H⋯O hydrogen bonds.

## Related literature

For the preparation of the title compound, see: Savitha & Gowda (2006 ▶). For our studies of the effect of substituents on the structures of *N*-(ar­yl)aryl­sulfonamides, see: Gowda *et al.* (2009**a* ▶,*b* ▶,c* ▶). For related structures, see: Gelbrich *et al.* (2007 ▶); Perlovich *et al.* (2006 ▶).
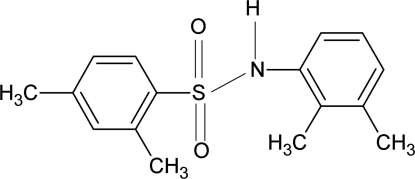


## Experimental

### 

#### Crystal data

C_16_H_19_NO_2_S
*M* *_r_* = 289.38
Triclinic, 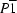

*a* = 8.3643 (7) Å
*b* = 10.975 (1) Å
*c* = 16.996 (2) Å
α = 83.034 (9)°
β = 80.100 (7)°
γ = 81.796 (9)°
*V* = 1513.7 (3) Å^3^
*Z* = 4
Mo *K*α radiation
μ = 0.22 mm^−1^
*T* = 299 K
0.34 × 0.30 × 0.20 mm

#### Data collection

Oxford Diffraction Xcalibur diffractometer with a Sapphire CCD detector
Absorption correction: multi-scan (*CrysAlis RED*; Oxford Diffraction, 2009 ▶) *T*_min_ = 0.931, *T*_max_ = 0.958
11164 measured reflections
6136 independent reflections
4196 reflections with *I* > 2σ(*I*)
*R*_int_ = 0.016

#### Refinement

*R*[*F* ^2^ > 2σ(*F* ^2^)] = 0.058
*wR*(*F* ^2^) = 0.161
*S* = 1.03
6136 reflections
375 parameters
H atoms treated by a mixture of independent and constrained refinement
Δρ_max_ = 0.66 e Å^−3^
Δρ_min_ = −0.41 e Å^−3^

### 

Data collection: *CrysAlis CCD* (Oxford Diffraction, 2009 ▶); cell refinement: *CrysAlis RED* (Oxford Diffraction, 2009 ▶); data reduction: *CrysAlis RED*; program(s) used to solve structure: *SHELXS97* (Sheldrick, 2008 ▶); program(s) used to refine structure: *SHELXL97* (Sheldrick, 2008 ▶); molecular graphics: *PLATON* (Spek, 2009 ▶); software used to prepare material for publication: *SHELXL97*.

## Supplementary Material

Crystal structure: contains datablocks I, global. DOI: 10.1107/S1600536810011669/ci5070sup1.cif

Structure factors: contains datablocks I. DOI: 10.1107/S1600536810011669/ci5070Isup2.hkl

Additional supplementary materials:  crystallographic information; 3D view; checkCIF report

## Figures and Tables

**Table 1:** Hydrogen-bond geometry (Å, °)

*D*—H⋯*A*	*D*—H	H⋯*A*	*D*⋯*A*	*D*—H⋯*A*
N1—H1*N*⋯O3	0.83 (3)	2.15 (3)	2.952 (3)	161 (3)
N2—H2*N*⋯O1	0.79 (3)	2.22 (3)	2.982 (3)	164 (3)

**Fig. 1.**
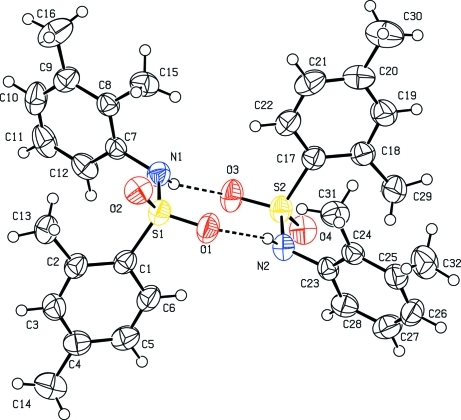
The two independent molecules of (I), showing the atom labelling scheme. Displacement ellipsoids are drawn at the 50% probability level. Dashed lines indicate hydrogen bonds.

**Fig. 2.**
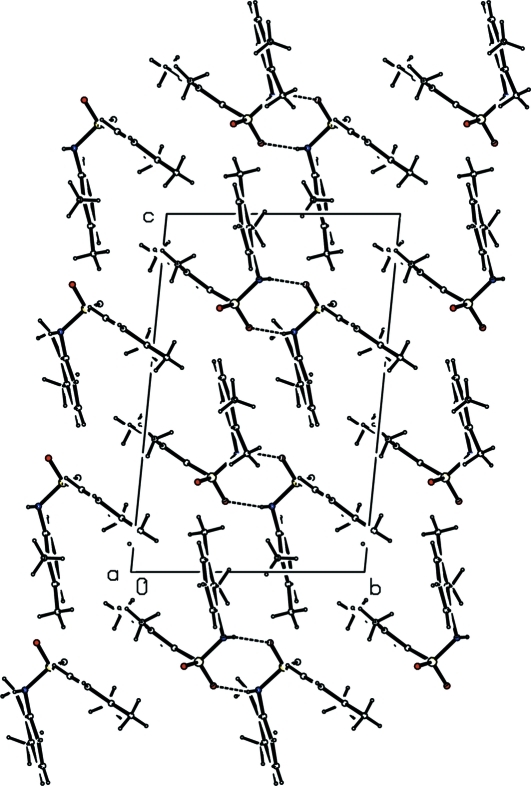
Molecular packing of (I) with hydrogen bonding shown as dashed lines.

## supplementary crystallographic information

### Comment

As part of a study of substituent effects on the structures of *N*-(aryl)arylsulfonamides (Gowda *et al.*, 2009*a,b,c*), in the present work, the structure of 2,4-dimethyl-*N*-(2,3-dimethylphenyl)benzenesulfonamide (I) has been determined (Fig. 1). The asymmetric unit contains two independent molecules. Both molecules are bent at the *N*-atoms with C—SO_2_—NH—C torsion angles of 70.1 (2) and -66.0 (2)°, compared to the values of 53.9 (2)° in 2,4-dimethyl-*N*-(3,5-dimethylphenyl)benzenesulfonamide (II) (Gowda *et al.*, 2009*c*), 71.0 (2)° in *N*-(2,3-dimethylphenyl)benzenesulfonamide (III) (Gowda *et al.*, 2009*a*), and 46.1 (3)° (glide image of molecule 1) and 47.7 (3)° (molecule 2) in the two independent molecules of 2,4-dimethyl-*N*-(phenyl)benzenesulfonamide (IV) (Gowda *et al.*, 2009*b*).

The sulfonyl and anilino benzene rings in the two molecules of (I) are tilted relative to each other by 41.5 (1) and 43.8 (1)° in (I), compared to the values of 82.1 (1)° in (II), 64.8 (1)° in (III), and 67.5 (1)° (molecule 1) and 72.9 (1)° (molecule 2) in the two independent molecules of (IV), The remaining bond parameters in (I) are similar to those observed in (II), (III), (IV) and other aryl sulfonamides (Perlovich *et al.*, 2006; Gelbrich *et al.*, 2007).

In the crystal structure, pairs of intermolecular N—H···O hydrogen bonds (Table 1) link the independent molecules to form dimers as shown in Fig. 1 and Fig.2.

### Experimental

The solution of *m*-xylene (10 ml) in chloroform (40 ml) was treated dropwise with chlorosulfonic acid (25 ml) at 273 K. After the initial evolution of hydrogen chloride subsided, the reaction mixture was brought to room temperature and poured into crushed ice in a beaker. The chloroform layer was separated, washed with cold water and allowed to evaporate slowly. The residual 2,4-dimethylbenzenesulfonylchloride was treated with a stoichiometric amount of 2,3-dimethylaniline and boiled for 10 min. The reaction mixture was then cooled to room temperature and added to ice cold water (100 ml). The resultant solid 2,4-dimethyl-*N*- (2,3-dimethylphenyl)benzenesulfonamide was filtered under suction and washed thoroughly with cold water. It was then recrystallized to constant melting point from dilute ethanol. The purity of the compound was checked and characterized by recording its infrared and NMR spectra (Savitha & Gowda, 2006). The single crystals used in X-ray diffraction studies were grown in ethanolic solution by slow evaporation at room temperature.

### Refinement

H atoms of the NH groups were located in a difference map and their positional parameters were refined [N–H = 0.79 (3)–0.83 (3) Å]. The other H atoms were positioned with idealized geometry using a riding model with C–H = 0.93–0.96 Å. All H atoms were refined with isotropic displacement parameters (set to 1.2 times of the U_eq_ of the parent atom).

### Crystal data

**Table:**

C_16_H_19_NO_2_S	*Z* = 4
*M**_r_* = 289.38	*F*(000) = 616
Triclinic, *P*1	*D*_x_ = 1.270 Mg m^−^^3^
Hall symbol: -P 1	Mo *K*α radiation, λ = 0.71073 Å
*a* = 8.3643 (7) Å	Cell parameters from 3007 reflections
*b* = 10.975 (1) Å	θ = 2.5–28.0°
*c* = 16.996 (2) Å	µ = 0.22 mm^−^^1^
α = 83.034 (9)°	*T* = 299 K
β = 80.100 (7)°	Prism, yellow
γ = 81.796 (9)°	0.34 × 0.30 × 0.20 mm
*V* = 1513.7 (3) Å^3^	

### Data collection

**Table:**

Oxford Diffraction Xcalibur diffractometer with a Sapphire CCD detector	6136 independent reflections
Radiation source: fine-focus sealed tube	4196 reflections with *I* > 2σ(*I*)
graphite	*R*_int_ = 0.016
Rotation method data acquisition using ω and φ scans	θ_max_ = 26.4°, θ_min_ = 2.5°
Absorption correction: multi-scan (*CrysAlis RED*; Oxford Diffraction, 2009)	*h* = −10→10
*T*_min_ = 0.931, *T*_max_ = 0.958	*k* = −13→13
11164 measured reflections	*l* = −21→21

### Refinement

**Table:**

Refinement on *F*^2^	Primary atom site location: structure-invariant direct methods
Least-squares matrix: full	Secondary atom site location: difference Fourier map
*R*[*F*^2^ > 2σ(*F*^2^)] = 0.058	Hydrogen site location: inferred from neighbouring sites
*wR*(*F*^2^) = 0.161	H atoms treated by a mixture of independent and constrained refinement
*S* = 1.03	*w* = 1/[σ^2^(*F*_o_^2^) + (0.0699*P*)^2^ + 0.9151*P*] where *P* = (*F*_o_^2^ + 2*F*_c_^2^)/3
6136 reflections	(Δ/σ)_max_ = 0.002
375 parameters	Δρ_max_ = 0.66 e Å^−^^3^
0 restraints	Δρ_min_ = −0.41 e Å^−^^3^

### Special details

**Table:**

Geometry. All esds (except the esd in the dihedral angle between two l.s. planes) are estimated using the full covariance matrix. The cell esds are taken into account individually in the estimation of esds in distances, angles and torsion angles; correlations between esds in cell parameters are only used when they are defined by crystal symmetry. An approximate (isotropic) treatment of cell esds is used for estimating esds involving l.s. planes.
Refinement. Refinement of *F*^2^ against ALL reflections. The weighted *R*-factor wR and goodness of fit *S* are based on *F*^2^, conventional *R*-factors *R* are based on *F*, with *F* set to zero for negative *F*^2^. The threshold expression of *F*^2^ > σ(*F*^2^) is used only for calculating *R*-factors(gt) etc. and is not relevant to the choice of reflections for refinement. *R*-factors based on *F*^2^ are statistically about twice as large as those based on *F*, and *R*- factors based on ALL data will be even larger.

### Fractional atomic coordinates and isotropic or equivalent isotropic displacement parameters (Å^2^)

**Table:**

	*x*	*y*	*z*	*U*_iso_*/*U*_eq_	
C1	0.7579 (3)	0.1917 (2)	0.30498 (15)	0.0439 (6)	
C2	0.8079 (3)	0.0826 (2)	0.35144 (17)	0.0487 (6)	
C3	0.6896 (4)	0.0046 (3)	0.38031 (18)	0.0559 (7)	
H3	0.7200	−0.0685	0.4110	0.067*	
C4	0.5295 (4)	0.0283 (3)	0.36652 (18)	0.0548 (7)	
C5	0.4848 (4)	0.1366 (3)	0.32071 (19)	0.0582 (8)	
H5	0.3781	0.1550	0.3101	0.070*	
C6	0.5965 (3)	0.2167 (3)	0.29087 (18)	0.0532 (7)	
H6	0.5642	0.2895	0.2605	0.064*	
C7	0.9403 (3)	0.3704 (2)	0.40309 (15)	0.0405 (6)	
C8	1.1020 (3)	0.3881 (2)	0.40397 (16)	0.0431 (6)	
C9	1.1659 (4)	0.3549 (3)	0.47568 (18)	0.0532 (7)	
C10	1.0649 (5)	0.3099 (3)	0.54282 (18)	0.0649 (9)	
H10	1.1067	0.2888	0.5906	0.078*	
C11	0.9048 (5)	0.2950 (3)	0.54161 (19)	0.0664 (9)	
H11	0.8397	0.2652	0.5880	0.080*	
C12	0.8422 (4)	0.3247 (3)	0.47111 (18)	0.0545 (7)	
H12	0.7346	0.3142	0.4692	0.065*	
C13	0.9783 (4)	0.0452 (3)	0.3710 (2)	0.0703 (9)	
H13A	1.0148	0.1136	0.3902	0.084*	
H13B	1.0510	0.0220	0.3235	0.084*	
H13C	0.9778	−0.0236	0.4116	0.084*	
C14	0.4087 (4)	−0.0607 (3)	0.4010 (2)	0.0770 (10)	
H14A	0.4628	−0.1313	0.4293	0.092*	
H14B	0.3640	−0.0869	0.3584	0.092*	
H14C	0.3221	−0.0206	0.4374	0.092*	
C15	1.2017 (4)	0.4467 (3)	0.33078 (19)	0.0594 (8)	
H15A	1.1299	0.4970	0.2980	0.071*	
H15B	1.2662	0.3833	0.3007	0.071*	
H15C	1.2725	0.4973	0.3469	0.071*	
C16	1.3387 (4)	0.3701 (4)	0.4809 (2)	0.0815 (11)	
H16A	1.3584	0.4534	0.4616	0.098*	
H16B	1.4123	0.3136	0.4486	0.098*	
H16C	1.3563	0.3531	0.5357	0.098*	
N1	0.8735 (3)	0.4042 (2)	0.32974 (14)	0.0487 (6)	
H1N	0.783 (4)	0.447 (3)	0.3348 (18)	0.058*	
O1	0.8196 (3)	0.37379 (19)	0.19753 (12)	0.0638 (6)	
O2	1.0524 (2)	0.24770 (19)	0.25134 (12)	0.0596 (5)	
S1	0.88758 (9)	0.30509 (6)	0.26408 (4)	0.0484 (2)	
C17	0.6666 (3)	0.7822 (2)	0.21219 (16)	0.0459 (6)	
C18	0.6232 (3)	0.8894 (2)	0.16309 (18)	0.0503 (7)	
C19	0.7455 (4)	0.9638 (3)	0.1334 (2)	0.0621 (8)	
H19	0.7199	1.0352	0.1002	0.075*	
C20	0.9028 (4)	0.9375 (3)	0.1505 (2)	0.0621 (8)	
C21	0.9409 (4)	0.8305 (3)	0.1997 (2)	0.0644 (9)	
H21	1.0461	0.8107	0.2122	0.077*	
C22	0.8243 (4)	0.7539 (3)	0.22987 (18)	0.0552 (7)	
H22	0.8512	0.6821	0.2625	0.066*	
C23	0.4896 (3)	0.6176 (2)	0.10779 (15)	0.0403 (6)	
C24	0.6025 (3)	0.6394 (2)	0.03794 (16)	0.0427 (6)	
C25	0.5393 (4)	0.6793 (2)	−0.03382 (17)	0.0523 (7)	
C26	0.3736 (4)	0.6952 (3)	−0.0335 (2)	0.0615 (8)	
H26	0.3337	0.7213	−0.0813	0.074*	
C27	0.2650 (4)	0.6736 (3)	0.0354 (2)	0.0628 (8)	
H27	0.1531	0.6862	0.0342	0.075*	
C28	0.3231 (3)	0.6332 (3)	0.10660 (18)	0.0526 (7)	
H28	0.2506	0.6166	0.1534	0.063*	
C29	0.4565 (4)	0.9307 (3)	0.1406 (2)	0.0710 (10)	
H29A	0.3803	0.9503	0.1878	0.085*	
H29B	0.4219	0.8656	0.1172	0.085*	
H29C	0.4609	1.0028	0.1026	0.085*	
C30	1.0290 (5)	1.0232 (4)	0.1153 (3)	0.0918 (12)	
H30A	1.0768	1.0470	0.1577	0.110*	
H30B	0.9775	1.0955	0.0875	0.110*	
H30C	1.1128	0.9813	0.0784	0.110*	
C31	0.7824 (3)	0.6221 (3)	0.03934 (18)	0.0588 (8)	
H31A	0.8049	0.5729	0.0878	0.071*	
H31B	0.8197	0.7014	0.0373	0.071*	
H31C	0.8382	0.5810	−0.0062	0.071*	
C32	0.6546 (5)	0.7030 (3)	−0.11119 (19)	0.0778 (10)	
H32A	0.7213	0.6273	−0.1243	0.093*	
H32B	0.7232	0.7631	−0.1049	0.093*	
H32C	0.5925	0.7337	−0.1536	0.093*	
N2	0.5434 (3)	0.5754 (2)	0.18338 (14)	0.0469 (5)	
H2N	0.627 (4)	0.532 (3)	0.1814 (18)	0.056*	
O3	0.5930 (3)	0.59831 (19)	0.31781 (12)	0.0651 (6)	
O4	0.3674 (2)	0.73252 (18)	0.26308 (12)	0.0575 (5)	
S2	0.53042 (9)	0.67152 (6)	0.25099 (4)	0.0487 (2)	

### Atomic displacement parameters (Å^2^)

**Table:**

	*U*^11^	*U*^22^	*U*^33^	*U*^12^	*U*^13^	*U*^23^
C1	0.0468 (15)	0.0455 (14)	0.0409 (15)	0.0010 (11)	−0.0121 (11)	−0.0104 (12)
C2	0.0506 (16)	0.0447 (14)	0.0516 (16)	0.0046 (12)	−0.0169 (13)	−0.0076 (12)
C3	0.0653 (19)	0.0428 (15)	0.0594 (19)	−0.0036 (13)	−0.0149 (15)	−0.0011 (13)
C4	0.0581 (18)	0.0546 (16)	0.0546 (18)	−0.0077 (14)	−0.0093 (14)	−0.0154 (14)
C5	0.0461 (16)	0.0651 (18)	0.066 (2)	−0.0035 (14)	−0.0159 (14)	−0.0128 (16)
C6	0.0520 (16)	0.0534 (16)	0.0555 (18)	0.0040 (13)	−0.0212 (13)	−0.0045 (13)
C7	0.0483 (15)	0.0314 (12)	0.0427 (14)	−0.0057 (10)	−0.0086 (11)	−0.0037 (10)
C8	0.0499 (15)	0.0370 (13)	0.0423 (15)	−0.0060 (11)	−0.0057 (11)	−0.0051 (11)
C9	0.0568 (17)	0.0502 (15)	0.0555 (18)	0.0025 (13)	−0.0174 (14)	−0.0156 (14)
C10	0.100 (3)	0.0516 (17)	0.0416 (17)	0.0086 (17)	−0.0207 (17)	−0.0049 (14)
C11	0.096 (3)	0.0519 (17)	0.0432 (18)	−0.0120 (17)	0.0093 (16)	0.0029 (14)
C12	0.0590 (18)	0.0488 (15)	0.0526 (18)	−0.0138 (13)	0.0055 (13)	−0.0047 (13)
C13	0.061 (2)	0.0565 (18)	0.094 (3)	0.0012 (15)	−0.0319 (18)	0.0124 (17)
C14	0.073 (2)	0.077 (2)	0.086 (3)	−0.0253 (18)	−0.0081 (19)	−0.010 (2)
C15	0.0515 (17)	0.0656 (18)	0.0616 (19)	−0.0195 (14)	−0.0001 (14)	−0.0060 (15)
C16	0.065 (2)	0.099 (3)	0.089 (3)	0.0086 (19)	−0.0332 (19)	−0.036 (2)
N1	0.0485 (13)	0.0439 (12)	0.0538 (14)	0.0008 (10)	−0.0144 (11)	−0.0041 (11)
O1	0.0778 (14)	0.0661 (13)	0.0476 (12)	−0.0039 (11)	−0.0223 (10)	0.0064 (10)
O2	0.0529 (12)	0.0671 (13)	0.0570 (13)	−0.0047 (10)	−0.0030 (9)	−0.0103 (10)
S1	0.0531 (4)	0.0499 (4)	0.0421 (4)	−0.0027 (3)	−0.0118 (3)	−0.0021 (3)
C17	0.0507 (16)	0.0436 (14)	0.0448 (15)	−0.0017 (12)	−0.0113 (12)	−0.0093 (12)
C18	0.0495 (16)	0.0418 (14)	0.0612 (18)	−0.0015 (12)	−0.0141 (13)	−0.0090 (13)
C19	0.064 (2)	0.0461 (16)	0.078 (2)	−0.0048 (14)	−0.0169 (16)	−0.0052 (15)
C20	0.0514 (18)	0.0637 (19)	0.075 (2)	−0.0111 (14)	−0.0056 (15)	−0.0245 (17)
C21	0.0495 (17)	0.074 (2)	0.075 (2)	0.0061 (15)	−0.0213 (16)	−0.0271 (18)
C22	0.0545 (17)	0.0564 (17)	0.0567 (18)	0.0027 (14)	−0.0185 (14)	−0.0110 (14)
C23	0.0491 (15)	0.0332 (12)	0.0379 (14)	−0.0036 (10)	−0.0058 (11)	−0.0042 (10)
C24	0.0499 (15)	0.0365 (12)	0.0429 (15)	−0.0088 (11)	−0.0066 (11)	−0.0058 (11)
C25	0.073 (2)	0.0433 (14)	0.0434 (16)	−0.0117 (13)	−0.0124 (14)	−0.0041 (12)
C26	0.080 (2)	0.0523 (17)	0.058 (2)	−0.0022 (15)	−0.0328 (17)	−0.0041 (14)
C27	0.0535 (18)	0.0624 (19)	0.079 (2)	−0.0035 (14)	−0.0242 (17)	−0.0161 (17)
C28	0.0459 (16)	0.0538 (16)	0.0580 (18)	−0.0101 (12)	−0.0029 (13)	−0.0086 (14)
C29	0.0588 (19)	0.0538 (18)	0.100 (3)	−0.0028 (15)	−0.0298 (18)	0.0126 (18)
C30	0.067 (2)	0.090 (3)	0.124 (4)	−0.025 (2)	−0.008 (2)	−0.024 (2)
C31	0.0499 (17)	0.0703 (19)	0.0556 (18)	−0.0144 (14)	0.0005 (13)	−0.0076 (15)
C32	0.109 (3)	0.077 (2)	0.0454 (19)	−0.022 (2)	−0.0035 (18)	0.0044 (17)
N2	0.0525 (14)	0.0437 (12)	0.0413 (13)	−0.0003 (10)	−0.0061 (11)	−0.0001 (10)
O3	0.0854 (15)	0.0675 (13)	0.0381 (11)	0.0013 (11)	−0.0128 (10)	0.0030 (10)
O4	0.0536 (12)	0.0617 (12)	0.0523 (12)	−0.0006 (9)	0.0027 (9)	−0.0089 (10)
S2	0.0574 (4)	0.0501 (4)	0.0362 (4)	−0.0020 (3)	−0.0062 (3)	−0.0025 (3)

### Geometric parameters (Å, °)

**Table:**

C1—C6	1.395 (4)	C17—C22	1.386 (4)
C1—C2	1.405 (4)	C17—C18	1.399 (4)
C1—S1	1.768 (3)	C17—S2	1.775 (3)
C2—C3	1.387 (4)	C18—C19	1.389 (4)
C2—C13	1.508 (4)	C18—C29	1.501 (4)
C3—C4	1.382 (4)	C19—C20	1.379 (4)
C3—H3	0.93	C19—H19	0.93
C4—C5	1.381 (4)	C20—C21	1.388 (4)
C4—C14	1.502 (4)	C20—C30	1.512 (5)
C5—C6	1.366 (4)	C21—C22	1.371 (4)
C5—H5	0.93	C21—H21	0.93
C6—H6	0.93	C22—H22	0.93
C7—C12	1.383 (4)	C23—C28	1.382 (4)
C7—C8	1.397 (3)	C23—C24	1.403 (4)
C7—N1	1.440 (3)	C23—N2	1.437 (3)
C8—C9	1.402 (4)	C24—C25	1.409 (4)
C8—C15	1.503 (4)	C24—C31	1.493 (4)
C9—C10	1.382 (4)	C25—C26	1.372 (4)
C9—C16	1.497 (4)	C25—C32	1.509 (4)
C10—C11	1.376 (5)	C26—C27	1.371 (5)
C10—H10	0.93	C26—H26	0.93
C11—C12	1.374 (4)	C27—C28	1.381 (4)
C11—H11	0.93	C27—H27	0.93
C12—H12	0.93	C28—H28	0.93
C13—H13A	0.96	C29—H29A	0.96
C13—H13B	0.96	C29—H29B	0.96
C13—H13C	0.96	C29—H29C	0.96
C14—H14A	0.96	C30—H30A	0.96
C14—H14B	0.96	C30—H30B	0.96
C14—H14C	0.96	C30—H30C	0.96
C15—H15A	0.96	C31—H31A	0.96
C15—H15B	0.96	C31—H31B	0.96
C15—H15C	0.96	C31—H31C	0.96
C16—H16A	0.96	C32—H32A	0.96
C16—H16B	0.96	C32—H32B	0.96
C16—H16C	0.96	C32—H32C	0.96
N1—S1	1.630 (2)	N2—S2	1.632 (2)
N1—H1N	0.83 (3)	N2—H2N	0.79 (3)
O1—S1	1.436 (2)	O3—S2	1.439 (2)
O2—S1	1.424 (2)	O4—S2	1.423 (2)
			
C6—C1—C2	119.8 (3)	C22—C17—C18	120.8 (3)
C6—C1—S1	116.4 (2)	C22—C17—S2	116.1 (2)
C2—C1—S1	123.8 (2)	C18—C17—S2	123.0 (2)
C3—C2—C1	116.4 (2)	C19—C18—C17	116.6 (3)
C3—C2—C13	118.8 (3)	C19—C18—C29	117.8 (3)
C1—C2—C13	124.8 (3)	C17—C18—C29	125.7 (3)
C4—C3—C2	124.3 (3)	C20—C19—C18	123.6 (3)
C4—C3—H3	117.9	C20—C19—H19	118.2
C2—C3—H3	117.9	C18—C19—H19	118.2
C5—C4—C3	117.7 (3)	C19—C20—C21	118.1 (3)
C5—C4—C14	121.5 (3)	C19—C20—C30	120.2 (3)
C3—C4—C14	120.8 (3)	C21—C20—C30	121.7 (3)
C6—C5—C4	120.3 (3)	C22—C21—C20	120.3 (3)
C6—C5—H5	119.8	C22—C21—H21	119.8
C4—C5—H5	119.8	C20—C21—H21	119.8
C5—C6—C1	121.5 (3)	C21—C22—C17	120.7 (3)
C5—C6—H6	119.3	C21—C22—H22	119.7
C1—C6—H6	119.3	C17—C22—H22	119.7
C12—C7—C8	121.8 (3)	C28—C23—C24	121.7 (2)
C12—C7—N1	119.5 (2)	C28—C23—N2	117.3 (2)
C8—C7—N1	118.7 (2)	C24—C23—N2	120.9 (2)
C7—C8—C9	118.3 (2)	C23—C24—C25	117.3 (2)
C7—C8—C15	120.5 (2)	C23—C24—C31	121.6 (2)
C9—C8—C15	121.1 (3)	C25—C24—C31	121.1 (3)
C10—C9—C8	118.7 (3)	C26—C25—C24	120.0 (3)
C10—C9—C16	120.1 (3)	C26—C25—C32	120.1 (3)
C8—C9—C16	121.2 (3)	C24—C25—C32	119.8 (3)
C11—C10—C9	122.5 (3)	C25—C26—C27	121.9 (3)
C11—C10—H10	118.8	C25—C26—H26	119.1
C9—C10—H10	118.8	C27—C26—H26	119.1
C12—C11—C10	119.2 (3)	C26—C27—C28	119.5 (3)
C12—C11—H11	120.4	C26—C27—H27	120.2
C10—C11—H11	120.4	C28—C27—H27	120.2
C11—C12—C7	119.5 (3)	C27—C28—C23	119.6 (3)
C11—C12—H12	120.2	C27—C28—H28	120.2
C7—C12—H12	120.2	C23—C28—H28	120.2
C2—C13—H13A	109.5	C18—C29—H29A	109.5
C2—C13—H13B	109.5	C18—C29—H29B	109.5
H13A—C13—H13B	109.5	H29A—C29—H29B	109.5
C2—C13—H13C	109.5	C18—C29—H29C	109.5
H13A—C13—H13C	109.5	H29A—C29—H29C	109.5
H13B—C13—H13C	109.5	H29B—C29—H29C	109.5
C4—C14—H14A	109.5	C20—C30—H30A	109.5
C4—C14—H14B	109.5	C20—C30—H30B	109.5
H14A—C14—H14B	109.5	H30A—C30—H30B	109.5
C4—C14—H14C	109.5	C20—C30—H30C	109.5
H14A—C14—H14C	109.5	H30A—C30—H30C	109.5
H14B—C14—H14C	109.5	H30B—C30—H30C	109.5
C8—C15—H15A	109.5	C24—C31—H31A	109.5
C8—C15—H15B	109.5	C24—C31—H31B	109.5
H15A—C15—H15B	109.5	H31A—C31—H31B	109.5
C8—C15—H15C	109.5	C24—C31—H31C	109.5
H15A—C15—H15C	109.5	H31A—C31—H31C	109.5
H15B—C15—H15C	109.5	H31B—C31—H31C	109.5
C9—C16—H16A	109.5	C25—C32—H32A	109.5
C9—C16—H16B	109.5	C25—C32—H32B	109.5
H16A—C16—H16B	109.5	H32A—C32—H32B	109.5
C9—C16—H16C	109.5	C25—C32—H32C	109.5
H16A—C16—H16C	109.5	H32A—C32—H32C	109.5
H16B—C16—H16C	109.5	H32B—C32—H32C	109.5
C7—N1—S1	121.05 (17)	C23—N2—S2	120.16 (17)
C7—N1—H1N	115 (2)	C23—N2—H2N	116 (2)
S1—N1—H1N	112 (2)	S2—N2—H2N	110 (2)
O2—S1—O1	119.10 (13)	O4—S2—O3	118.80 (13)
O2—S1—N1	107.81 (12)	O4—S2—N2	108.11 (12)
O1—S1—N1	105.08 (12)	O3—S2—N2	104.97 (12)
O2—S1—C1	109.11 (12)	O4—S2—C17	109.21 (12)
O1—S1—C1	107.12 (13)	O3—S2—C17	107.64 (13)
N1—S1—C1	108.16 (12)	N2—S2—C17	107.58 (12)
			
C6—C1—C2—C3	0.5 (4)	C22—C17—C18—C19	0.3 (4)
S1—C1—C2—C3	178.7 (2)	S2—C17—C18—C19	−176.3 (2)
C6—C1—C2—C13	−179.9 (3)	C22—C17—C18—C29	−179.1 (3)
S1—C1—C2—C13	−1.8 (4)	S2—C17—C18—C29	4.2 (4)
C1—C2—C3—C4	−0.4 (4)	C17—C18—C19—C20	−0.6 (5)
C13—C2—C3—C4	−180.0 (3)	C29—C18—C19—C20	178.9 (3)
C2—C3—C4—C5	0.3 (4)	C18—C19—C20—C21	0.3 (5)
C2—C3—C4—C14	−179.2 (3)	C18—C19—C20—C30	179.7 (3)
C3—C4—C5—C6	−0.3 (4)	C19—C20—C21—C22	0.1 (5)
C14—C4—C5—C6	179.2 (3)	C30—C20—C21—C22	−179.2 (3)
C4—C5—C6—C1	0.5 (4)	C20—C21—C22—C17	−0.4 (5)
C2—C1—C6—C5	−0.6 (4)	C18—C17—C22—C21	0.1 (4)
S1—C1—C6—C5	−178.9 (2)	S2—C17—C22—C21	177.0 (2)
C12—C7—C8—C9	1.9 (4)	C28—C23—C24—C25	0.6 (4)
N1—C7—C8—C9	180.0 (2)	N2—C23—C24—C25	179.3 (2)
C12—C7—C8—C15	−175.4 (2)	C28—C23—C24—C31	−179.8 (2)
N1—C7—C8—C15	2.7 (4)	N2—C23—C24—C31	−1.2 (4)
C7—C8—C9—C10	−2.0 (4)	C23—C24—C25—C26	−0.1 (4)
C15—C8—C9—C10	175.2 (3)	C31—C24—C25—C26	−179.6 (3)
C7—C8—C9—C16	179.4 (3)	C23—C24—C25—C32	−179.3 (3)
C15—C8—C9—C16	−3.4 (4)	C31—C24—C25—C32	1.1 (4)
C8—C9—C10—C11	0.9 (4)	C24—C25—C26—C27	0.2 (4)
C16—C9—C10—C11	179.4 (3)	C32—C25—C26—C27	179.5 (3)
C9—C10—C11—C12	0.6 (5)	C25—C26—C27—C28	−0.9 (5)
C10—C11—C12—C7	−0.8 (4)	C26—C27—C28—C23	1.4 (4)
C8—C7—C12—C11	−0.4 (4)	C24—C23—C28—C27	−1.3 (4)
N1—C7—C12—C11	−178.5 (2)	N2—C23—C28—C27	180.0 (2)
C12—C7—N1—S1	−91.8 (3)	C28—C23—N2—S2	−77.9 (3)
C8—C7—N1—S1	90.0 (3)	C24—C23—N2—S2	103.4 (2)
C7—N1—S1—O2	−47.8 (2)	C23—N2—S2—O4	51.8 (2)
C7—N1—S1—O1	−175.8 (2)	C23—N2—S2—O3	179.5 (2)
C7—N1—S1—C1	70.1 (2)	C23—N2—S2—C17	−66.0 (2)
C6—C1—S1—O2	−154.2 (2)	C22—C17—S2—O4	152.1 (2)
C2—C1—S1—O2	27.5 (3)	C18—C17—S2—O4	−31.1 (3)
C6—C1—S1—O1	−24.0 (2)	C22—C17—S2—O3	21.9 (2)
C2—C1—S1—O1	157.7 (2)	C18—C17—S2—O3	−161.3 (2)
C6—C1—S1—N1	88.8 (2)	C22—C17—S2—N2	−90.8 (2)
C2—C1—S1—N1	−89.5 (2)	C18—C17—S2—N2	86.0 (2)

### Hydrogen-bond geometry (Å, °)

**Table:**

*D*—H···*A*	*D*—H	H···*A*	*D*···*A*	*D*—H···*A*
N1—H1N···O3	0.83 (3)	2.15 (3)	2.952 (3)	161 (3)
N2—H2N···O1	0.79 (3)	2.22 (3)	2.982 (3)	164 (3)
